# Neutrophils at the Maternal‐Fetal Interface: Agents of Protection or Destruction?

**DOI:** 10.1111/aji.70181

**Published:** 2025-10-30

**Authors:** Sallie L. Fell, Sydney M. Nemphos, James E. Prusak, Amitinder Kaur, Jamie O. Lo, Jennifer A. Manuzak

**Affiliations:** ^1^ Division of Immunology Tulane National Biomedical Research Center Covington Louisiana USA; ^2^ Department of Microbiology and Immunology Tulane University School of Medicine New Orleans Louisiana USA; ^3^ Division of Maternal‐Fetal Medicine Department of Obstetrics and Gynecology Oregon Health and Science University Portland Oregon USA; ^4^ Division of Reproductive and Developmental Sciences Oregon National Primate Research Center Oregon Health and Science University Beaverton Oregon USA; ^5^ Department of Tropical Medicine and Infectious Disease Tulane University Celia Scott Weatherhead School of Public Health and Tropical Medicine New Orleans Louisiana USA

**Keywords:** bacterial infections, decidua, extracellular traps, innate immunity, neutrophils, neutrophil activation, parasitic diseases, placenta, viral diseases

## Abstract

Neutrophils, traditionally recognized for their role in innate immunity, have emerged as a key cell population at the maternal‐fetal interface, during both uncomplicated and pathological pregnancies. Neutrophil effector functions, including phagocytosis, neutrophil extracellular trap formation, and degranulation, can play protective roles, such as preventing infection and facilitating tissue remodeling during pregnancy. However, these effector functions may also contribute to excessive inflammation, tissue damage, and adverse pregnancy outcomes in the context of sterile inflammation or maternal infection, underscoring the dual nature of neutrophils at the maternal‐fetal interface. In this review, we examine the paradoxical nature of neutrophils at the maternal‐fetal interface. Further, the protective and deleterious roles of neutrophils during pregnancy are evaluated in the context of bacterial, viral, and parasitic infections. Insights from this review are anticipated to inform basic and clinical research aimed at identifying neutrophils or neutrophil components as biomarkers and therapeutic targets in obstetric conditions and infectious diseases during pregnancy.

## 1 Introduction

Pregnancy represents a unique immunological state that requires a delicate balance between maternal and fetal tolerogenic immune responses and necessary defense against pathogens. These processes require significant physiological alterations mediated by specialized cells at the maternal‐fetal interface. Neutrophils, key players in innate immunity, have multifaceted roles in the immunological landscape of pregnancy. These cells participate in essential pregnancy‐associated processes, including placental vascularization and tissue remodeling, and defend against infection. However, excessive neutrophil responses at the maternal‐fetal interface can result in inflammation and tissue damage, which contributes to adverse pregnancy outcomes, including preeclampsia, preterm birth, and pregnancy loss. Importantly, understanding the specific mechanisms through which neutrophil functions impact pregnancy immunomodulatory processes will inform future clinical research strategies. For example, a better understanding of neutrophil dynamics in pregnancy could support development of innate immune‐targeting therapies to prevent complications like preeclampsia, preterm birth, or chorioamnionitis, ultimately improving pregnancy outcomes.

In this review, we describe how neutrophils contribute to physiological processes required for normal pregnancies, including placental vascularization, cervical ripening, and postpartum tissue repair. We also describe how neutrophil activity can result in deleterious pregnancy outcomes, including preeclampsia, chorioamnionitis, and pregnancy loss, even in the absence of infectious stimuli. Finally, we discuss the role of neutrophils in the context of infection during pregnancy, with an emphasis on bacterial, viral, and parasitic pathogens.

## 2 Effector Functions of Neutrophils

Neutrophils, which constitute up to 70% of circulating leukocytes, are critical in the initial host defense against invading pathogens [1, 2]. The primary mechanisms through which neutrophils aid in the host immune response include: (1) phagocytosis; (2) generation of reactive oxygen species (ROS); (3) release of granules containing anti‐microbial peptides and enzymes; and (4) neutrophil extracellular trap (NET) formation, or the release of DNA decorated with granules that trap and eliminate pathogens [1, 2, 3, 4, 5, 6]. In Table 1, we provide an overview of the principal neutrophil effector functions to facilitate discussion of the role of neutrophils at the maternal‐fetal interface.

**TABLE 1:** Overview of principal neutrophil effector functions and their involvement in pathology.

Neutrophil effector function	Description of function	Involvement in pathology	References
Phagocytosis	Process by which neutrophils detect and engulf pathogens, apoptotic host cells, and cellular debris for clearance Particle uptake triggers phagosome maturation, in which the phagocytic vacuole and cytosolic granules fuse together	Dysregulation of neutrophil phagocytosis may result in unnecessary release of ROS and granule contents, potentially exacerbating tissue injury	Naish, 2023 [7] Jaumouillé, 2020 [8] Mayadas, 2014 [9] Sheppard, 2005 [10] Hirahashi, 2006 [11] DeLeo, 2020 [12]
Reactive Oxygen Species (ROS) Production	Potent antimicrobial mechanism by which NADPH oxidase catalyzes production of superoxide anion (O_2_^•−^), which can be converted into other toxic ROS including hydrogen peroxide (H_2_O_2_) and hypochlorous acid (HOCl) Neutrophil‐derived ROS can promote release of granule components, NET production, and stimulation of pro‐inflammatory cytokine production	Excessive release of these highly reactive molecules can paradoxically result in extracellular matrix damage and tissue necrosis	Mayadas, 2014 [9] Sheppard, 2005 [10] Winterbourn, 2016 [13] Lambeth, 2004 [14] Naik, 2011 [15] Sheshachalam, 2014 [16] Mittal, 2014 [17] Lu, 2022 [18] Chen, 2010 [19]
Degranulation	Process by which neutrophils release contents of intracellular granules to kill microbes Neutrophil granules are categorized by the components within the granules: Secretory granules: contain plasma proteins and Fc and complement receptors Tertiary granules: contain matrix metalloproteases (MMPs) Secondary granules: contain lactoferrin and other enzymes Primary granules: require greatest stimulus for release and contain most pro‐inflammatory mediators including myeloperoxidase (MPO), defensins, neutrophil elastase (NE), and azurocidin	Unregulated neutrophil degranulation can lead to excessive inflammation and immune activation, contributing to collateral tissue damage	Lacy, 2006 [20] Eichelberger, 2020 [21] Herrero‐Cervera, 2022 [22]
Neutrophil Extracellular Trap (NET) Formation	Also called NETosis, a mechanism by which neutrophils excrete web‐like DNA structure decorated with antimicrobial granule components which trap and kill pathogens Two main types of NETosis are vital NETosis (neutrophils “survive” NET formation) and suicidal NETosis (NET formation occurs while neutrophils undergo cell death) Vital NETosis is mediated through Toll‐like receptors (TLRs), which activate PAD4, a protein that facilitates hypercitrullination of nuclear histones Suicidal NETosis is mediated through induction of NADPH oxidase following protein kinase C activation via ERK‐MEK signaling pathway	NETosis is largely considered pro‐inflammatory, but suicidal NETosis is viewed as more inflammatory than vital NETosis due to disintegration of membranes and subsequent release of ROS and granule components Uncontrolled NET formation is associated with excessive inflammation and subsequent pathology	Remijsen, 2011 [23] Papayannopoulos, 2009 [24] Tan, 2021 [25] Fuchs, 2007 [26] Huang, 2020 [27] Kessenbrock, 2009 [28] Knight, 2012 [29] Hakkim, 2010 [30] Rodríguez‐Espinosa, 2015 [31] Burgener, 2020 [32] Yipp, 2012 [33] Pieterse, 2016 [34] Douda, 2015 [35] Naffah de Souza, 2017 [36]

## 3 Neutrophils at the Maternal‐Fetal Interface

### 3.1 Recruitment and Function of Neutrophils at the Maternal‐Fetal Interface

The precise role of neutrophils in reproductive immunology remains incompletely understood [37], due in part to the fact that they comprise a relatively small portion of immune cells in the human decidua [38]. Specifically, low frequencies of neutrophils are detected in the first trimester (∼5% of CD45+ cells), followed by a gradual increase throughout gestation, and finally an influx of neutrophils in the decidua and myometrium at parturition [38, 39]. Neutrophil migration to the decidua is partially characterized and is likely driven by chemoattraction to CXCL8 (IL‐8) and granulocyte‐macrophage colony‐stimulating factor (GM‐CSF) secreted by endometrial epithelial cells [40] or Group 3 decidual innate lymphoid cells (ILC3) [41]. Placenta‐derived factors may also contribute to neutrophil recruitment, such as syncytiotrophoblast microparticles, which can activate neutrophils [42]. Further, placental galectins increase CXCL8 production by T cells, suggesting a role in neutrophil migration to the placenta [43].

### 3.2 Physiological Role of Neutrophils in Pregnancy

**FIGURE 1:**
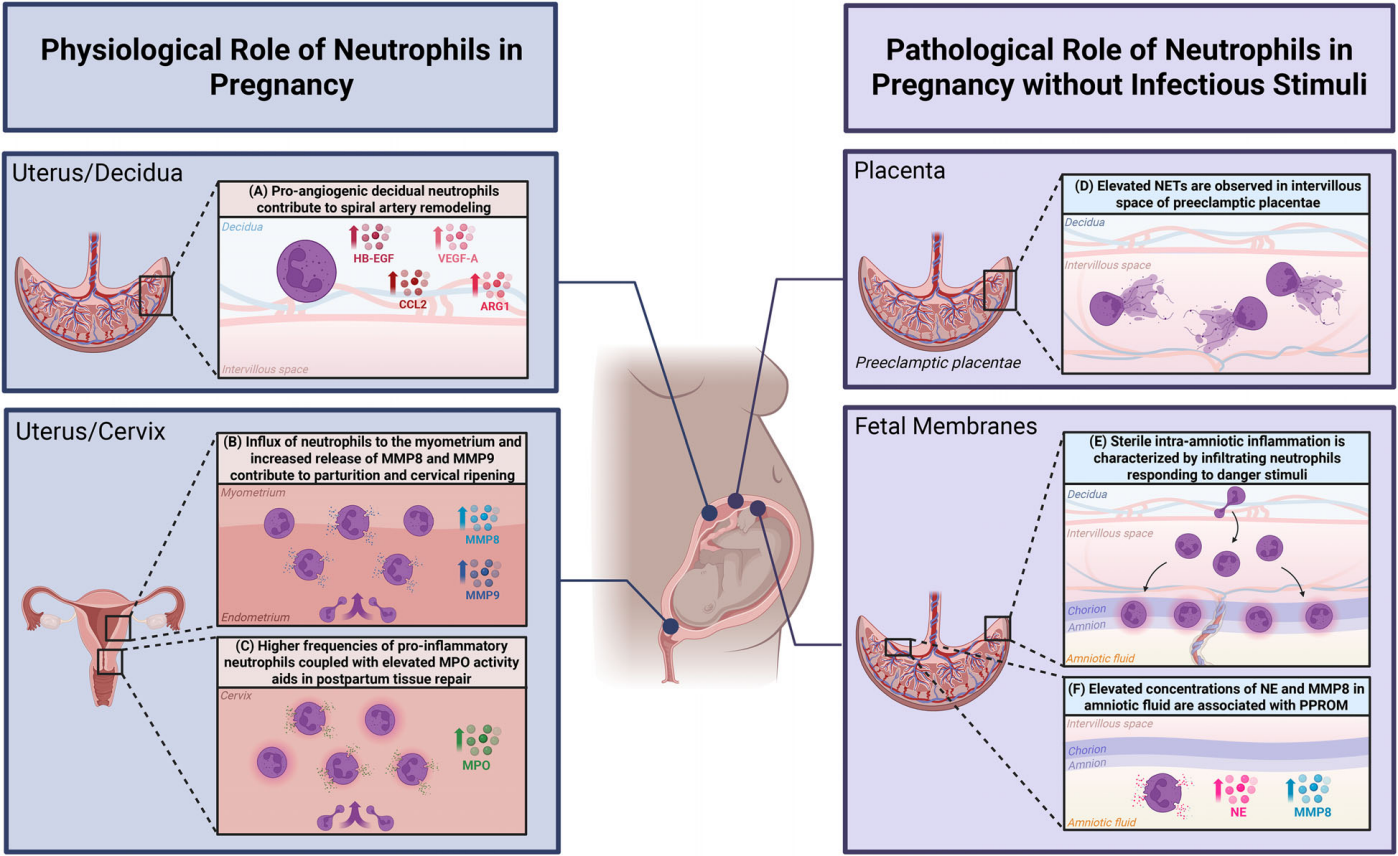
The physiological and pathological roles of neutrophils in pregnancy. (A) Decidual neutrophils demonstrate a distinct pro‐angiogenic phenotype, which highlights the role of these cells in spiral artery remodeling. Specifically, these cells have a marked increase in expression of heparin binding‐epidermal growth factor (HB‐EGF), vascular endothelial growth factor (VEGF‐A), C‐C motif chemokine ligand 2 (CCL2), and arginase‐1 (ARG1). (B) Cervical ripening is often accompanied by an influx of neutrophils to the myometrium. These infiltrating neutrophils produce MMP8 and MMP9, which aid in extracellular collagen degradation. (C) Higher frequencies of neutrophils with pro‐inflammatory gene expression and elevated MPO activity have been observed in the postpartum cervix, suggesting a role in postpartum tissue repair. (D) Neutrophils have also been implicated in adverse pregnancy outcomes, including preeclampsia pathology. Specifically, increased markers of NET formation have been observed in preeclamptic placentae compared to normotensive placentae. (E) Sterile intra‐amniotic inflammation, which occurs in the absence of infection, is also characterized by an influx of neutrophils to the chorioamniotic membrane in response to non‐infectious danger signals. (F) Preterm prelabor rupture of membranes (PPROM) is also associated with an elevated concentration of MMP8 and NE in the amniotic fluid, suggesting an adverse role of neutrophils in this context. This figure was created with BioRender.

Recent research has revealed that neutrophils play a critical, albeit less well characterized, role in supporting physiological processes necessary for successful pregnancies [44]. Here, we review the mechanisms by which neutrophils positively impact pregnancy outcomes, including placentation [44, 45], parturition [46], and cervical remodeling [46, 47, 48] (Figure 1A–C).

#### 3.2.1 Placental Vascularization

During pregnancy, uterine spiral arteries are remodeled into highly dilated vessels to maximize blood flow to the placenta and provide oxygen and nutrients to the developing fetus [49, 50]. Spiral artery remodeling begins soon after blastocyst implantation [51]. Continued uterine vasculature remodeling occurs throughout gestation [52, 53], likely to meet the increasing demands of the developing fetus [54]. Angiogenesis, the formation of new blood vessels from existing ones [55], also enhances blood circulation to the fetus by forming a dense network of new vasculature [56, 57]. This multi‐step process begins with a local or systemic increase in angiogenic factors, including vascular endothelial growth factor (VEGF) and placental growth factor (PlGF), followed by the breakdown of the endothelial basement membrane to enable migration and proliferation of endothelial cells [58]. Taken together, spiral artery remodeling and angiogenesis are critical processes to facilitate successful placental vascularization.

There is a significant increase in neutrophils migrating and localizing near spiral arteries in the human decidua basalis from 6 to 20 weeks of gestation [54]. Additionally, a significant absence of spiral artery remodeling and defective decidual neutrophil recruitment was observed in *Rag2^−/−^IL2rg^−/−^* mice, which lack decidual NK (dNK) cells [54, 59], suggesting that interactions between neutrophils and dNK cells are critical for uterine vascular remodeling. Indeed, dNK cells secrete IL‐8 at different stages of pregnancy, including implantation, spiral artery remodeling, trophoblast invasion, and parturition [60]. Whether dNK secretion of IL‐8 is directly linked with neutrophil infiltration in this context remains to be elucidated. Other studies have examined decidual neutrophil expression of heparin binding‐epidermal growth factor (HB‐EGF) [41], which is a critical signaling molecule for extravillous trophoblast invasion and spiral artery remodeling [61, 62]. GM‐CSF derived from an ILC subpopulation, natural cytotoxicity receptor (NCR)+ ILC3s, induced elevated expression of HB‐EGF in decidual neutrophils in vitro, suggesting that crosstalk between these cell subsets may enhance the angiogenic nature of decidual neutrophils [41]. Indeed, decidual neutrophils have a distinct pro‐angiogenic phenotype, with elevated expression of VEGF‐A, arginase‐1 (ARG1), and C‐C motif chemokine ligand 2 (CCL2) [54] (Figure 1A). Moreover, decidual neutrophils were shown to induce a regulatory T cell phenotype in a unique subpopulation of decidual T cells called neutrophil‐induced T (niT) cells, which are angiogenic and necessary for placental vascularization [63]. Taken together, neutrophils can directly or indirectly promote placental vascularization during pregnancy.

#### 3.2.2 Parturition and Cervical Ripening in Term Labor

Spontaneous labor at term is associated with a “parturition cascade” that involves an influx of immune cells to the cervix, coupled with the release of pro‐inflammatory mediators [39, 64]. Both macrophages and neutrophils have been implicated in this process [65]. Cervical ripening, the physiological process of softening and dilation of the cervix in preparation for vaginal delivery, is accompanied by an influx of neutrophils to the myometrium [64, 66] (Figure 1B). Neutrophils are a rich source of collagenase during labor, namely MMP‐8 and MMP‐9, which reduces the collagen‐dependent rigidity of the cervix and supports their role in extracellular collagen degradation and cervical ripening [67, 68, 69, 70].

Conversely, some studies have challenged the contribution of neutrophils to labor‐related cervical modifications. Increased frequencies of macrophages and neutrophils have been observed after vaginal delivery, but only macrophage frequencies were increased prior to labor onset [71]. The contribution of neutrophils to cervical ripening has also been investigated using steroid 5α‐reductase type 1 null mice (*Srd5a1* ^−/−^), which fail to undergo cervical ripening due to insufficient local progesterone metabolism [72]. This study demonstrated that neutrophil depletion had no significant effect on parturition timing or success. The authors postulated that neutrophil infiltration was therefore not required for initiation of cervical ripening and instead contributed to postpartum cervical remodeling [72].

#### 3.2.3 Postpartum Tissue Repair

Traditionally, inflammation of the myometrium has been considered a driver in the onset of term labor [73, 74]. However, some studies suggest that inflammation could instead be a consequence of labor induction, designed to prepare the uterus for postpartum remodeling [75, 76]. The role of neutrophils in postpartum tissue repair has been recently investigated [77, 78], although their contribution in this context remains incompletely understood [79]. Postpartum repair is characterized by increased pro‐inflammatory gene expression and higher frequencies of neutrophils [80], which may help clear cellular debris, like proteoglycan fragments and damaged collagen [81] (Figure 1C). Prior studies have observed elevated MPO activity in the cervix of postpartum sheep and mice, suggesting neutrophil infiltration of cervical tissue in the postpartum period [72, 82]. A clinical study in humans reported significantly elevated markers of neutrophil degranulation and NET formation in the periphery during the early postpartum period (48 h after delivery) [79], suggesting a protective role against postpartum infections and complications.

### 3.3 Pathological Role of Neutrophils in Pregnancy Without Infectious Stimuli

Despite the critical role of neutrophils in multiple pregnancy processes, these cells have often been considered harmful in the context of pregnancy [44]. In the following sections, we outline the role of neutrophils in non‐infection‐driven adverse pregnancy outcomes, including preeclampsia [83], preterm labor [84], and fetal loss [38] (Figure 1D – F).

#### 3.3.1 Preeclampsia

Preeclampsia, a hypertensive disorder of pregnancy, affects 5%–7% of all pregnancies and accounts for 70 000 maternal deaths and 500 000 fetal deaths worldwide annually [85]. Despite its large impact on maternal and fetal morbidity and mortality, the precise mechanisms underlying preeclampsia remain incompletely understood [86]. Current research suggests that preeclampsia pathophysiology is multifactorial and that immune cells have etiological roles, including neutrophils [83] (Figure 1D). For example, circulating neutrophil counts are significantly higher in patients with severe compared to mild preeclampsia or normotensive patients [87], suggesting that peripheral neutrophil counts correlate with preeclampsia severity. Additionally, peripheral blood neutrophils are activated in preeclampsia [88] and cause endothelial dysfunction by infiltrating systemic vasculature and releasing ROS, TNF‐α, and MPO [89, 90, 91]. Alterations in neutrophil effector functions have also been noted during preeclamptic pregnancies. One study observed that plasma levels of NE, indicative of degranulation and NET formation, are elevated in preeclamptic compared to normotensive patients [92]. Additionally, large numbers of NETs have been observed in the intervillous space of preeclamptic placentae [42]. Efficient activation and NET formation were demonstrated by neutrophils following in vitro stimulation with placenta‐derived IL‐8 and syncytiotrophoblast microparticles [42]. However, it remains unclear whether NETs are a triggering event for preeclampsia or are the result of other underlying placental inflammatory events [93]. Nonetheless, the excessive presence of NETs in preeclamptic placentae and induction of NET formation by placenta‐derived microparticles and cytokines suggests a role for these elements in preeclampsia [93, 94].

#### 3.3.2 Preterm Labor and Sterile Intra‐Amniotic Inflammation

Neutrophils play a critical role in the pathogenesis of acute histologic chorioamnionitis by infiltrating the chorioamniotic membrane [95]. This condition may be caused by intra‐amniotic infection or, more frequently, by “sterile” intra‐amniotic inflammation, which occurs in the absence of infection [96, 97] (Figure 1E). In sterile intra‐amniotic inflammation, the inflammatory milieu is exacerbated by infiltrating neutrophils responding to non‐infectious danger signals resulting from trauma, ischemia‐reperfusion injury, urate crystals, or chemical injuries [97, 98]. Additionally, prior work has shown that preterm prelabor rupture of membranes (PPROM) is associated with a significant increase in amniotic fluid concentration of MMP‐8 and NE [99, 100], indicating an association between inflammatory neutrophil functions and PPROM (Figure 1F). Taken together, neutrophil‐mediated inflammation heightens the risk of preterm labor, fetal inflammation, and perinatal mortality.

#### 3.3.3 Recurrent Pregnancy Loss

Recurrent pregnancy loss (RPL) is defined as two or more consecutive failed clinical pregnancies confirmed by ultrasound or histopathology [101]. Nearly half of RPL cases lack a clear etiology [102]. One potential immune etiology of RPL is antiphospholipid antibody syndrome (APS) [93, 103], an autoimmune thrombophilic disorder in which circulating antibodies recognize and attack phospholipid‐binding proteins rather than phospholipid itself [104]. Prior work has demonstrated that APS leads to activation of the complement cascade, which can be deleterious to the developing fetus [105, 106]. More recent work has speculated that APS‐associated complement activation may result in neutrophil activation and NET formation [93]. A previous study using the mildly hypertensive BPH/5 mouse model demonstrated that complement activation recruits neutrophils to the maternal‐fetal interface, resulting in elevated local TNF‐α, reduced VEGF, abnormal placentation, and subsequent fetal death [107]. Pregnancies were rescued with complement blockade, neutrophil depletion, or TNF‐α blockade [107]. Taken together, current evidence points to a relationship between complement activation and neutrophil recruitment and activity at the maternal‐fetal interface, potentially enhancing the risk for fetal loss. However, more work is warranted to examine neutrophil infiltration and activity as underlying mechanisms of RPL.

### 3.4 Role of Neutrophils in Pregnancy With Infectious Stimuli

Thus far, we have described how neutrophil effector functions are necessary for successful pregnancies but when overactive may exacerbate risk for pregnancy pathologies. Next, we review current clinical and experimental evidence regarding the role of neutrophils during various infections in pregnancy. We first examine bacterial pathogens, including *Listeria monocytogenes*, Group B Streptococcus, and *Chlamydia trachomatis*, which are associated with adverse outcomes during pregnancy. Next, we discuss viral pathogens including the TORCH pathogens (Rubella virus, cytomegalovirus, and herpes simplex virus) as well as other clinically relevant viruses (human immunodeficiency virus, zika virus, SARS‐CoV‐2) that have been implicated in pregnancy complications. Finally, we examine parasitic pathogens including the TORCH pathogen *Toxoplasma gondii* as well as *Plasmodium falciparum*, both of which can detrimentally impact maternal and fetal health.

#### 3.4.1 Bacterial Pathogens

##### 3.4.1.1 *Listeria monocytogenes* (LM)

LM is a facultative anaerobic, gram‐positive bacillus that is the causative agent of listeriosis, a rare but severe foodborne illness [108]. LM can cross the intestinal barrier, disseminate in the bloodstream, and eventually cross the placental barrier [109]. At the maternal‐fetal interface, LM infection can result in adverse sequelae, including chorioamnionitis, preterm labor, neonatal sepsis, and neonatal death [110].

**FIGURE 2:**
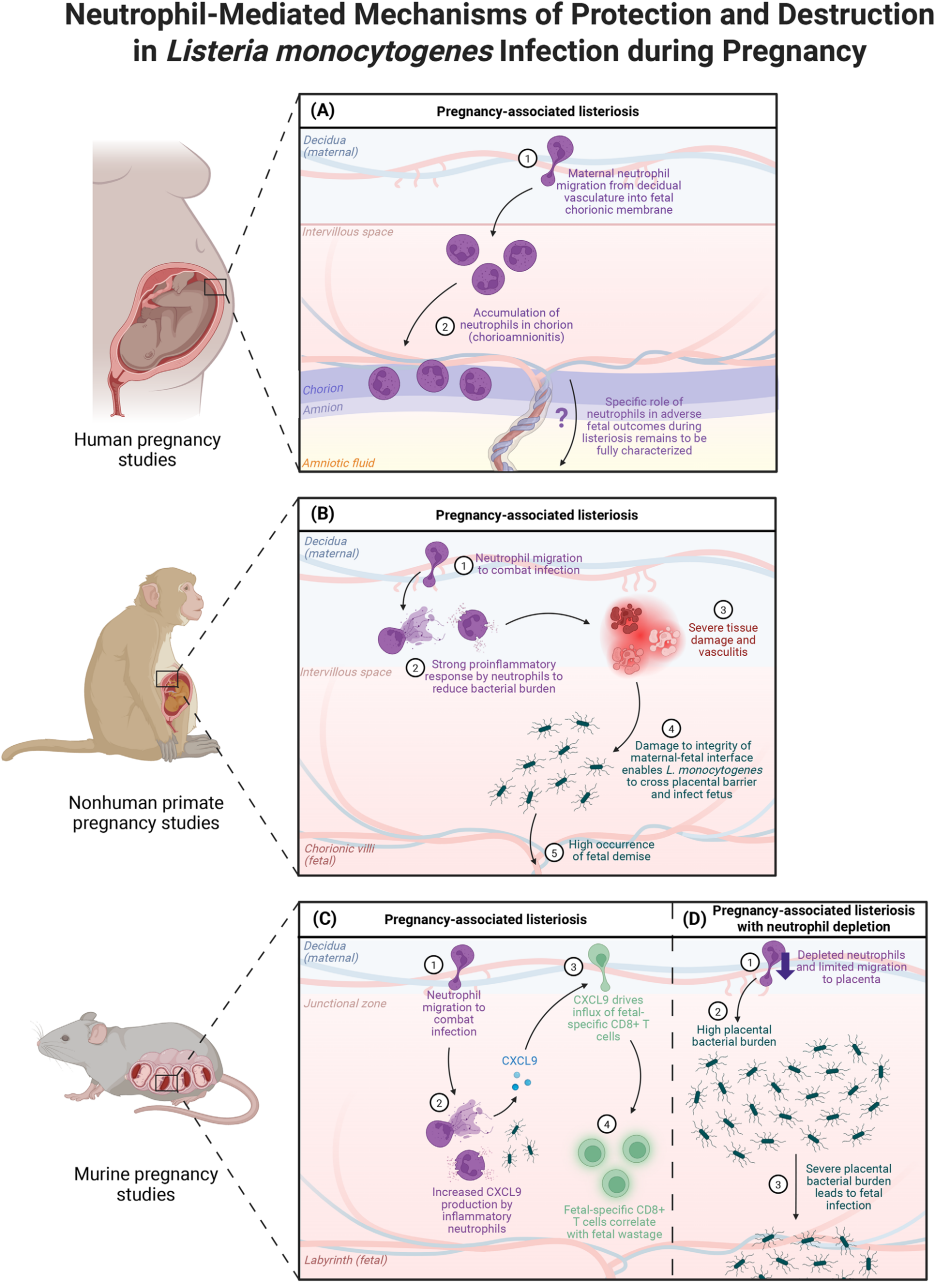
Neutrophil‐mediated mechanisms of protection and destruction in *Listeria monocytogenes* infection during pregnancy. (A) Studies from human patients with *Listeria monocytogenes* (LM) infection during pregnancy commonly report a clinical observation of chorioamnionitis, or infiltration of neutrophils in the chorionic and amniotic membranes. Human pregnancies complicated by LM infection often result in adverse outcomes, including preterm labor, neonatal sepsis, and fetal death. (B) A study in LM‐infected pregnant nonhuman primates (NHPs) reported substantial neutrophil infiltration and vasculitis in umbilical cord and placental tissues. The authors speculated that LM infection elicits a strong pro‐inflammatory response by neutrophils in the NHP decidua that combats bacterial burden but, conversely, results in severe tissue damage, subsequently resulting in fetal demise. (C) Murine studies have shed light on the mechanisms underlying the pathology of LM infection in pregnancy. In a murine model of pregnancy‐associated listeriosis, increased CXCL9‐producing neutrophils were observed in the placenta. The elevated levels of CXCL9 correlated with an influx of fetal‐specific CD8+ T cells into the decidua, which coincided with increased fetal wastage in the mice. (D) In a different murine model of pregnancy‐associated listeriosis, pregnant mice were depleted of polymorphonuclear cells, including neutrophils and eosinophils, to investigate the role of these cells in LM placental invasion and fetal infection. The neutrophil‐depleted mice demonstrated a significantly higher bacterial burden in the placenta and fetus when compared to nontreated pregnant controls. This study demonstrates the importance of neutrophils in controlling bacterial spread and fetal infection during listeriosis in pregnancy. This figure was created with BioRender.

Prior work has emphasized the role of macrophages during listeriosis in pregnancy, as bacterial replication occurs in these cells [111]. However, neutrophils are also an essential component of the innate immune response during listeriosis in pregnancy (Figure 2). Pregnancy‐associated listeriosis is characterized by an influx of neutrophils into the maternal‐fetal interface [112, 113]. Indeed, a murine study showed that by day 2–3 post‐LM infection, there was a significant increase in bacterial burden in placentae and fetuses of neutrophil‐depleted mice compared to controls, indicating that neutrophils are crucial in controlling bacterial spread and fetal infection after disruption of the maternal‐fetal interface [112].

Although protective against bacterial burden, aberrant neutrophil responses can lead to excessive inflammation and tissue damage. Recently, 60% of cases of pregnancy‐associated listeriosis were demonstrated to have placental histopathology with neutrophil infiltration, consistent with acute chorioamnionitis [114]. A murine model of LM in pregnancy observed increased CXCL9‐producing inflammatory neutrophils in the placenta that were associated with an influx of fetal‐specific CD8+ T cells into the decidua. These findings coincided with, but may not directly underlie, the fetal wastage reported in these mice [115]. Further, umbilical cord and placental tissue from LM‐infected cynomolgus macaques demonstrated neutrophilic infiltration and vasculitis, indicating that LM elicits a strong pro‐inflammatory response in the nonhuman primate decidua that resolves infection but results in severe tissue damage at the maternal‐fetal interface [116]. Taken together, while neutrophils protect against maternal bacterial burden during pregnancy‐associated listeriosis, excessive neutrophil responses may disrupt the integrity of the maternal‐fetal interface, leading to subsequent fetal infection and demise.

##### 3.4.1.2 Group B Streptococcus (GBS)

*Streptococcus agalactiae* (GBS) is a commensal gram‐positive bacterial species that asymptomatically colonizes approximately 18% of pregnant adults [117]. GBS can ascend the reproductive tract during pregnancy, resulting in adverse outcomes such as stillbirth, chorioamnionitis, preterm birth, and neonatal sepsis and meningitis [118].

Neutrophils have been previously characterized as protective against GBS infection [119, 120], and there is growing evidence for a similar protective role of neutrophils during GBS infection in pregnancy. Significant neutrophil infiltration into placental and decidual tissues was reported in a murine model of ascending GBS infection in pregnancy [121]. In this study, elevated NET formation was observed in the GBS‐infected group compared to the control [121]. These findings are consistent with prior work on GBS‐infected human tissues, which exhibit abundant neutrophilic infiltrates and GBS‐induced NET formation [121]. This study also reported that NET‐associated lactoferrin was highly antimicrobial, inhibiting GBS growth and viability in vitro [121]. Interestingly, immune evasion mechanisms exhibited by GBS have been characterized. Specifically, GBS DNase enables liberation of bacteria from murine NETs [122], and hyaluronidase‐expressing GBS is capable of dampening neutrophil ROS production in pregnant rhesus macaques [123]. Taken together, neutrophil recruitment, along with NET formation, is important for protection against GBS infection in pregnancy.

##### 3.4.1.3 Chlamydia trachomatis

*Chlamydia trachomatis* is the gram‐negative, anaerobic, obligate intracellular bacterium responsible for causing chlamydia [124]. Globally, *C. trachomatis* is the most common bacterial sexually transmitted infection [125]. This pathogen can ascend the female reproductive tract and infect the placenta and amniotic fluid [126]. Chlamydia in pregnancy has been associated with spontaneous abortion, chorioamnionitis, PPROM, and stillbirth [127].

It is well established that neutrophils are recruited to sites of chlamydial infection, but the precise role of neutrophils in defense against this bacterium remains only partially understood [128]. In mice, neutrophils were shown to play a critical role in controlling early stages of *C*. *trachomatis* [128]. There is also growing evidence that *C. trachomatis* can evade neutrophil effector functions, including NET formation and phagocytosis [129, 130], but these immune evasion tactics have yet to be examined in the context of pregnancy. Further work is required to examine the protective or pro‐inflammatory effect of neutrophils recruited to the maternal‐fetal interface during *C. trachomatis* infection in pregnancy.

#### 3.4.2 Viral Pathogens

##### 3.4.2.1 Rubella Virus

Rubella virus is an enveloped, single‐stranded RNA virus capable of infecting microglia in developing human brains [131]. Rubella virus is one of the TORCH pathogens (*T. gondii*, other agents, Rubella virus, cytomegalovirus, and herpesvirus), which is a group of pathogens capable of evading maternal‐fetal immune responses, crossing the placental barrier, and infecting the fetus [132]. Rubella virus infection during pregnancy can result in congenital rubella syndrome (CRS), which causes cataract development, cardiac abnormalities, and sensorineural deafness in offspring [133]. With the help of effective vaccines and enhanced surveillance, global cases of CRS have significantly declined [134]. The pathogenesis of CRS remains poorly understood [135], but several mechanisms, including necrosis of chorionic villi, direct viral damage of infected cells by apoptosis, and cytopathic damage to endothelial cells, have been proposed [136]. Neutrophils were shown to release superoxide anion after stimulation with rubella viral antigen‐antibody complex in vitro [137], but complementary in vivo or ex vivo mechanistic studies of the role of neutrophils in congenital rubella are lacking.

##### 3.4.2.2 Cytomegalovirus (CMV)

CMV is another TORCH pathogen and is a member of the *Herpesviridae* family of viruses; subfamily Betaherpesvirinae. Congenital CMV (cCMV) infection is the leading infectious cause of hearing loss and developmental delays in children [138]. Neutrophils play a multifaceted role during CMV infection, including mediation of virus dissemination and exacerbation of CMV disease progression [139]. Neutrophil accumulation in tissues is common in immunocompromised hosts with CMV, including retinitis, pneumonitis, and central nervous system complications [139, 140, 141, 142, 143]. In a murine model of chronic CMV infection, persistent enhancement of neutrophil oxidative burst was observed even after resolution of acute infection [144]. While these studies shed light on the relationship between CMV and neutrophil biology, there remains a paucity of data on neutrophil responses during cCMV at the maternal‐fetal interface.

##### 3.4.2.3 Herpes Simplex Virus (HSV)

HSV is a double‐stranded DNA virus in the *Herpesviridae* family; subfamily Alphaherpesvirinae, with HSV type 1 (HSV1) and type 2 (HSV2) in humans [145]. HSV1 and HSV2 are both TORCH pathogens, as they are capable of transplacental transmission to the fetus, but transmission through a virus‐shedding lesion in the genital tract during vaginal delivery is a more common route of vertical transmission [146]. The acquisition of genital HSV during pregnancy is associated with spontaneous abortion, intrauterine growth restriction, preterm labor, and congenital and neonatal HSV infection [147]. While neutrophil responses to HSV have been examined to a limited extent in in vitro and in vivo studies [148, 149, 150, 151], there remain major knowledge gaps on the neutrophil response during genital HSV infection in pregnancy.

##### 3.4.2.4 Human Immunodeficiency Virus (HIV)

HIV is the causative agent of acquired immunodeficiency syndrome (AIDS). HIV is a member of the *Lentivirus* genus, part of the *Retroviridae* family. It is estimated that 1.3 million people with HIV (PWH) become pregnant each year [152]. Vertical HIV transmission can occur during pregnancy, labor and delivery, or breastfeeding [153]. Without adequate intervention, vertical transmission rates range from 15%–45% [154]. Even with antiretroviral therapy (ART), pregnant PWH have higher instances of miscarriage/stillbirth, preterm delivery, and low birthweight infants compared to their HIV‐negative counterparts [155].

There are limited studies examining the mechanisms underlying adverse pregnancy outcomes in pregnant PWH [156]. Altered angiogenesis in pregnant PWH has been investigated as a potential mechanism for preterm birth, small for gestational age infants, and stillbirth [156]. This study only examined the kinetics of angiogenic factors in pregnant PWH; it lacked a control group of HIV‐uninfected pregnant people. While the study presented detailed kinetics of angiogenic factors during pregnancy, the authors emphasized the need for additional studies to delineate the role of HIV infection in driving dysregulated angiogenesis in pregnancy.

**FIGURE 3:**
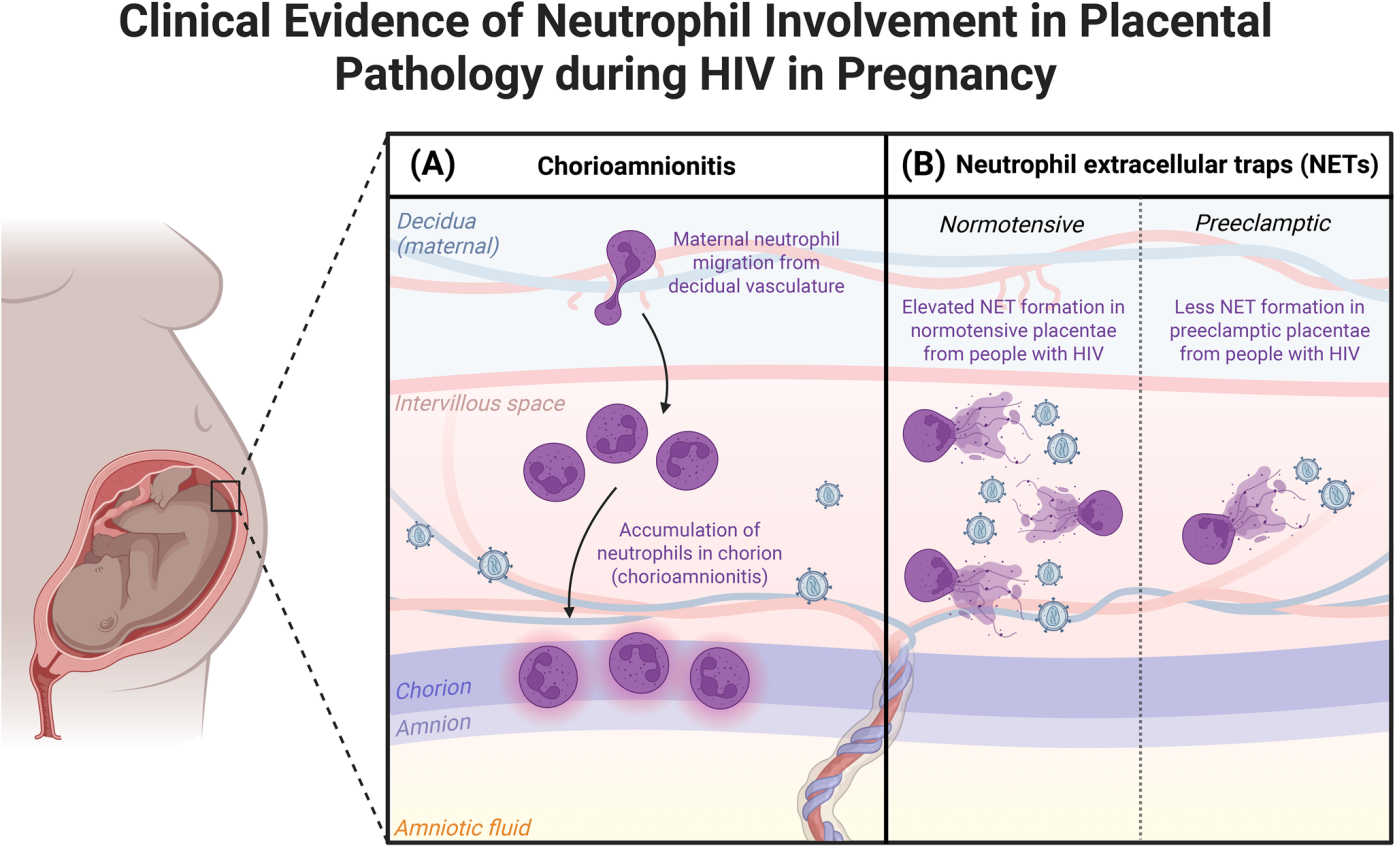
Clinical evidence of neutrophil involvement in placental pathology during HIV in pregnancy. (A) The most widely reported histopathological diagnosis in placentae from pregnant people with HIV (PWH) is acute chorioamnionitis, even in the context of antiretroviral therapy (ART). Histologic acute chorioamnionitis is characterized by neutrophil infiltration in the chorioamniotic membranes. (B) Elevated levels of neutrophil extracellular traps (NETs) have also been observed in placentae from PWH compared to those without HIV. Interestingly, pregnancy status (normotensive vs. preeclamptic) has an effect on NET formation in PWH. Specifically, normotensive PWH have increased NET formation compared to PWH with preeclampsia. This figure was created with BioRender.

Histologic acute chorioamnionitis, characterized by neutrophil infiltration in the chorioamniotic layers, is the most widely reported histopathological diagnosis in placentae from ART‐treated pregnant PWH (Figure 3) [157, 158]. The presence of NETs in placentae of PWH has also been examined as a driver of pathology (Figure 3). Histone H2A, a biomarker of NET formation, has been observed in normotensive and preeclamptic placentae from people with and without HIV [159]. This study observed significantly higher H2A expression in the intervillous space of placentae from PWH compared to those without HIV, indicating elevated NET formation. The authors suggest that the increased NET formation in the placentae of PWH may prevent vertical HIV transmission, as NETs can trap and eliminate HIV in vitro [160]. Interestingly, this study reported that placentae of preeclamptic PWH have decreased NETs compared to those from normotensive PWH [159]. Thus, pregnancy status (normotensive vs. preeclamptic) must be considered when examining the effects of HIV status on NET formation in placentae. A potential explanation for this phenomenon was proposed in a recent study [161], which showed that preeclampsia and HIV separately result in decreased placental reverse trans‐migration (r‐TM) of neutrophils from tissue back into circulation, where their pro‐inflammatory state may favor NET formation [78]. Taken together, while NETs may have virucidal effects against HIV, further work is necessary to unravel the role of neutrophils in placental angiogenesis in PWH.

##### 3.4.2.5 Zika Virus (ZIKV)

ZIKV is a vector‐borne RNA virus belonging to the genus *Flavivirus*, and ZIKV infection in pregnancy can result in teratogenic effects [162, 163]. Congenital Zika syndrome (CZS) is a result of vertical transmission of ZIKV and ultimately manifests as impaired central nervous system development, microcephaly, and spontaneous abortion [164, 165, 166]. In a rhesus macaque model of congenital ZIKV infection, neutrophil accumulation was observed in the decidua, placenta, and fetal membranes [167]. This accumulation did not appear to drive severe fetal manifestations, nor did it prevent vertical ZIKV transmission [167]. Neutrophilic chorioamnionitis has been observed in full‐term placentae collected from human patients infected with ZIKV during pregnancy [168]. Indeed, transcriptomic analyses demonstrated enriched gene sets associated with neutrophil function in a full‐term human placenta from a patient infected with ZIKV in first trimester compared to an uninfected control placenta [169]. Proteomic analyses of amniotic fluid revealed markers of neutrophil dysfunction in pregnant ZIKV‐infected patients [170]. Additionally, a marked decrease in proteins associated with neutrophil degranulation was observed in the amniotic fluid of ZIKV‐infected patients with microcephalic fetuses compared to ZIKV‐infected patients without pregnancies complicated by fetal microcephaly [170]. Taken together, these results indicate that disruption of neutrophil responses may enhance the risk of CZS occurrence [170].

##### 3.4.2.6 SARS‐CoV‐2

Coronavirus disease 2019 (COVID‐19), caused by severe acute respiratory syndrome coronavirus‐2 (SARS‐CoV‐2), is marked by symptoms ranging from mild respiratory issues to severe pneumonia and acute respiratory distress syndrome [171]. SARS‐CoV‐2 infection during pregnancy can result in placental insufficiency, preterm birth, and stillbirth [172, 173]. Vertical transmission of SARS‐CoV‐2 is extremely rare, as only three reports have demonstrated direct viral infection of placentae [174, 175, 176]. Prior work shows peripheral blood neutrophil frequencies were elevated in pregnant individuals infected with SARS‐CoV‐2 compared to nonpregnant individuals [177, 178]. Outside of cellular inflammation observed in placentae in case reports, current evidence does not implicate neutrophils in adverse pregnancy outcomes secondary to SARS‐CoV‐2 infection [179, 180].

#### 3.4.3 Parasitic Pathogens

##### 3.4.3.1 Toxoplasma gondii

*Toxoplasma gondii* is an obligate intracellular parasite that causes toxoplasmosis in immunocompromised or pregnant individuals [181]. *T. gondii* is a TORCH pathogen, and toxoplasmosis during pregnancy is associated with adverse outcomes, including miscarriage, stillbirth, preterm birth, congenital infection, hydrocephalus, and eye disease [182, 183]. The precise mechanisms underlying severe disease manifestations of toxoplasmosis in pregnancy remain incompletely understood [184, 185, 186, 187, 188].

Neutrophils help control *T. gondii* infection through the release of cytokines, ROS, and NETs [189, 190, 191, 192, 193, 194]. Additionally, neutrophil‐depleted nonpregnant mice demonstrate increased parasite burden and lethality, highlighting the importance of neutrophils in *T. gondii* infection [195]. In the context of congenital toxoplasmosis, ex vivo *T. gondii* infection of human placental chorionic villi explants resulted in increased secretion of IL‐8, which aids in neutrophil recruitment and activation [196]. Although IL‐8 is protective against *T. gondii* parasite burden, increased placental IL‐8 concentrations correlate with adverse outcomes, including congenital infection, preterm birth, and fetal loss [197, 198]. It is unclear whether neutrophil presence at the maternal‐fetal interface during toxoplasmosis is protective against parasite burden or detrimentally associated with adverse pregnancy outcomes [199].

##### 3.4.3.2 Plasmodium falciparum

Unicellular protozoan parasites in the *Plasmodium* genus are the causative agents of malaria [200]. Of the five *Plasmodium* spp. infective to humans, *Plasmodium falciparum* is responsible for the majority of malaria‐associated morbidity and mortality [201]. *P. falciparum* malaria in pregnancy (MIP) results in adverse maternal and fetal outcomes, including severe maternal anemia [202], preterm birth [203], low birthweight [204], and stillbirth [205]. Within the placental microenvironment, *P. falciparum* MIP can result in local inflammation [206], sequestration of infected red blood cells (iRBCs) [207], and immune cell infiltration [208].

Multiple studies have observed extensive intervillous inflammation with detrimental neutrophil accumulation in the placentae of *P. falciparum*‐infected patients [209, 210, 211]. Stimulation of syncytiotrophoblast‐like BeWo cells with *P. falciparum*‐iRBCs induced IL‐8 production in vitro, suggesting a mechanism for neutrophil recruitment to the placenta during MIP [210]. Similarly, elevated levels of IL‐8 are observed in the placental plasma of patients with placental malaria compared to uninfected placentae [212]. In this study, however, placental levels of IL‐8 did not correlate with neutrophil density by histological examination [212].

**FIGURE 4:**
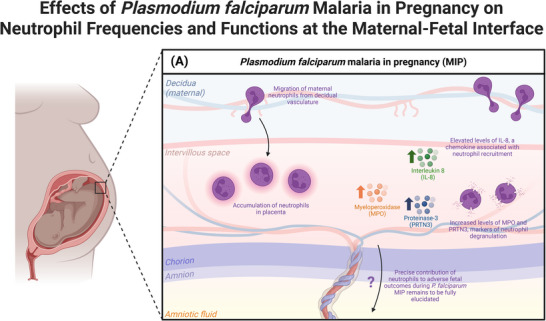
Neutrophil‐mediated mechanisms of protection and destruction in the context of *Plasmodium falciparum* malaria in pregnancy. (A) Multiple studies in humans have demonstrated a detrimental accumulation of neutrophils in the placentae of patients with *P. falciparum* infection. Additionally, elevated levels of interleukin‐8 (IL‐8), myeloperoxidase (MPO), and proteinase‐3 (PRTN3) have been observed in placental blood from patients with placental malaria. However, the precise role of neutrophils toward adverse fetal outcomes during *P. falciparum* malaria in pregnancy remains to be fully elucidated. This figure was created with BioRender.

The roles of neutrophil effector functions during MIP remain partially understood (Figure 4). On one hand, lower serum levels of MPO and NE have been observed in the periphery of patients with MIP [213]. Conversely, significantly elevated levels of MPO and proteinase 3 (PRTN3), indicators of neutrophil degranulation, were observed in the placental blood of patients with malaria, and both positively correlated with placental parasitemia, suggesting that neutrophil activation may correlate with infection severity [214]. Additionally, NET‐like structures were observed in the intervillous space of placentae from patients with malaria, although there was not association between malaria status and evidence of NETs [214]. Taken together, these findings do not distinguish the role of neutrophils in MIP as definitively protective or pathologic.

## 4 Conclusions and Future Directions

Despite the classical belief that neutrophils only mediate rapid and nonspecific immune responses, recent evidence has highlighted the multifaceted roles neutrophils play during pregnancy. Indeed, although neutrophils comprise a relatively small proportion of the immune cells at the maternal‐fetal interface, they contribute to multiple physiological processes during pregnancy, including placental vascularization, cervical modifications, and postpartum tissue repair. However, excessive neutrophil effector functions at the maternal‐fetal interface can result in adverse pregnancy outcomes. Inflammatory neutrophil processes, including degranulation, NET formation, and ROS production, must be tightly regulated to maintain homeostasis and ensure successful pregnancy.

Several novel approaches to therapeutically target neutrophils have been developed recently [215]. Neutrophil‐targeted therapeutic strategies may focus on enhancing, inhibiting, or restoring neutrophil functions [215]. More specifically, key therapeutic approaches to modulate neutrophil function may include targeting neutrophil development and production [216], inhibiting neutrophil accumulation at sites of infection or inflammation [217, 218, 219, 220], or reducing the adverse effects of neutrophil‐derived products like NE, MPO, or NETs [221, 222, 223]. These neutrophil‐targeted therapeutic approaches have been examined in a range of conditions, including arthritis [216, 217], chronic pulmonary obstructive disease (COPD) [219], and cystic fibrosis [224]. However, future research is warranted to examine the therapeutic potential of such neutrophil‐targeted approaches in the context of pregnancy.

In summary, future investigations are warranted to improve our understanding of the mechanisms of neutrophil‐mediated protection and immunopathology during pregnancy. Specifically, there are major knowledge gaps on the role of neutrophils at the maternal‐fetal interface in the context of rubella virus, CMV, HSV, SARS‐CoV‐2, and *C. trachomatis*. Although slightly more defined, further work is also needed to enhance our understanding of neutrophils in the immunopathology of *T. gondii*, malaria, ZIKV, HIV, GBS, and listeriosis in pregnancy. It is important to prioritize studies examining the recruitment of neutrophils and triggers of neutrophil activation at the maternal‐fetal interface. Ultimately, advancing our understanding of the multidimensional role of neutrophils during pregnancy will lay the foundation for the development of neutrophil‐targeted therapies and biomarkers in the context of pregnancy, which could enhance surveillance and help prevent complications, including preeclampsia, chorioamnionitis, and RPL, thereby improving pregnancy outcomes.

## Ethics Statement

The authors confirm that the ethical policies of the journal, as outlined on the guidelines page, have been followed. No ethical approval was required for this manuscript, as this is a review article with no original research data.

## Conflicts of Interest

The authors declare no conflicts of interest.

## Data Availability

Data sharing not applicable to this article as no datasets were generated or analyzed during the current study.
